# Hierarchical Co–Pi Clusters/Fe_2_O_3_ Nanorods/FTO Micropillars 3D Branched Photoanode for High-Performance Photoelectrochemical Water Splitting

**DOI:** 10.3390/nano12203664

**Published:** 2022-10-18

**Authors:** Nakhyun Kim, Sucheol Ju, Jisung Ha, Hojung Choi, Hansang Sung, Heon Lee

**Affiliations:** 1 Department of Semiconductor Systems Engineering, Korea University, Anam-ro 145, Sungbuk-gu, Seoul 136-701, Korea; 2 Department of Materials Science and Engineering, Korea University, Anam-ro 145, Sungbuk-gu, Seoul 136-701, Korea

**Keywords:** photoelectrochemical water splitting, hematite, direct printing, patterned fluorine-doped tin oxide

## Abstract

In this study, an efficient hierarchical Co–Pi cluster/Fe_2_O_3_ nanorod/fluorine-doped tin oxide (FTO) micropillar three-dimensional (3D) branched photoanode was designed for enhanced photoelectrochemical performance. A periodic array of FTO micropillars, which acts as a highly conductive “host” framework for uniform light scattering and provides an extremely enlarged active area, was fabricated by direct printing and mist-chemical vapor deposition (CVD). Fe_2_O_3_ nanorods that act as light absorber “guest” materials and Co–Pi clusters that give rise to random light scattering were synthesized via a hydrothermal reaction and photoassisted electrodeposition, respectively. The hierarchical 3D branched photoanode exhibited enhanced light absorption efficiency because of multiple light scattering, which was a combination of uniform light scattering from the periodic FTO micropillars and random light scattering from the Fe_2_O_3_ nanorods. Additionally, the large surface area of the 3D FTO micropillar, together with the surface area provided by the one-dimensional Fe_2_O_3_ nanorods, contributed to a remarkable increase in the specific area of the photoanode. Because of these enhancements and further improvements facilitated by decoration with a Co–Pi catalyst that enhanced water oxidation, the 3D branched Fe_2_O_3_ photoanode achieved a photocurrent density of 1.51 mA cm^−2^ at 1.23 V_RHE_, which was 5.2 times higher than that generated by the non-decorated flat Fe_2_O_3_ photoanode.

## 1. Introduction

With the increased focus on replacing fossil fuels with renewable energy sources, photoelectrochemical (PEC) water splitting has gained significant attention for the conversion of solar energy into clean chemical energy (H_2_) [1,2,3]. For efficient solar energy conversion, a PEC cell must absorb visible light, transport and separate the charge carriers, and transfer the separated charge carriers to the semiconductor/electrolyte surface [4,5,6]. However, dense photoelectrode films are limited by their low PEC efficiencies due to their small surface area and high reflectivity. To solve this problem and obtain increased PEC efficiency, many studies using micro-nanostructured photoelectrodes have been conducted [7,8]. It was shown that micro-nanostructured photoanodes can successfully decrease the carrier diffusion path, induce a light-scattering effect, and increase the surface area, thereby favoring a higher PEC efficiency.

To fabricate such structured photoanodes, hierarchical guest/host structures are commonly constructed [9,10]. Hierarchical guest/host type photoelectrodes can maximize the PEC efficiency by combining the effects of 1D, 2D, and 3D micro-nanostructures on light absorption and scattering, and by providing an extremely large specific surface area. In these hierarchical structures, the guest material must have a narrow bandgap and adequate band edge alignments to act as a photoactive layer that absorbs visible light and efficiently creates photogenerated charges for PEC reactions [11,12]. Metal oxides such as Fe_2_O_3_, BiVO_4_, and TiO_2_ are good photoactive guest materials, and they have often been investigated in studies on micro-nanostructured photoelectrodes. Since the host material serves as a growth scaffold for the guest material, host materials must exhibit high electrical conductivity and chemical resistance [13,14]. Because of its chemical stability, high optical transmittance, and low electrical resistance, fluorine-doped tin oxide (FTO) is deemed the most effective candidate for the host material. Many studies have employed guest-host-structured photoanodes using FTO as the host, such as FTO inverse opal/CdS nanorods [15], FTO inverse opal/TiO_2_ [16], and nanocone arrays of FTO/α-Fe_2_O_3_ [17] photoanodes. However, the processes used for the fabrication of the FTO framework in the above studies were complicated and could not easily fabricate uniform FTO over a large area. Therefore, it is necessary to develop new methods for the scalable fabrication of FTO-based micro-nanostructured photoanodes that are suitable for large-scale production and use.

In this study, a hierarchical Co–Pi cluster/Fe_2_O_3_ nanorod/FTO micropillar 3D branched photoanode was fabricated. Periodic FTO micropillars were constructed by performing direct printing and mist-chemical vapor deposition (CVD), which enable simple, low-cost, and large-area fabrication. Thereafter, 1D Fe_2_O_3_ nanorods were hydrothermally synthesized on the surface of the periodic FTO micropillars. The periodic FTO micropillars, which acted as a conductive framework, collected electrons to facilitate uniform light scattering and provide a large surface area for Fe_2_O_3_ nanorods to grow. Moreover, the Fe_2_O_3_ nanorods enabled random light scattering and increased the surface area further. Thus, the hierarchical Fe_2_O_3_ nanorod/FTO micropillar structure induced multiple light scattering mechanisms for enhanced light-harvesting efficiency. Thereafter, the Fe_2_O_3_ nanorods were supplemented with Co–Pi clusters to increase the surface oxygen evolution reaction (OER) and PEC performance of the entire photoanode. In terms of the photocurrent density (1.51 mA cm^−2^ at 1.23 V_RHE_), the hierarchical Co–Pi cluster/Fe_2_O_3_ nanorod/FTO micropillar 3D branched photoanode exhibited significant improvement compared to that of Fe_2_O_3_ nanorod/flat FTO.

## 2. Materials and Methods

### 2.1. Fabrication of Periodic FTO Micropillars

**Scheme 1:**
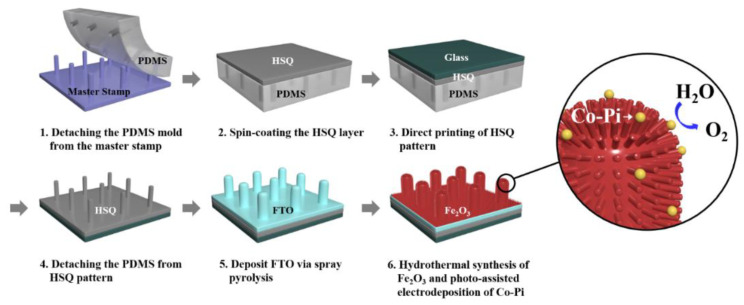
The fabrication process of FTO-M/Fe_2_O_3_/Co–Pi.

Scheme 1 illustrates the fabrication process for obtaining a composite photoanode comprising Co–Pi-decorated Fe_2_O_3_ nanorods on periodic FTO micropillars. Periodic FTO micropillars, labeled FTO-M, were fabricated using a process reported in earlier studies [18,19]. Polydimethylsiloxane (PDMS) was prepared using Sylgard 184A and Sylgard 184B (10:1 volume ratio). Thereafter, PDMS was applied to the photolithography-fabricated micropillar-array Si wafer. The PDMS mold was hardened (80 °C, 3 h) and then peeled from the micropillar-array Si wafer, producing a PDMS mold with a reverse micropillar structure. A hydrogen silsesquioxane (HSQ) solution was diluted in isobutyl methyl ketone (IBMK) and spread onto the PDMS mold (3000 rpm, 30 s), using the spin-coating method. Thereafter, an Eagle XG glass substrate (Corning) showing excellent acidity, alkalinity, and heat resistance, was applied to the PDMS mold at a pressure of 200 kPa for 1 min. After the detachment of the PDMS mold, an HSQ micropillar array was formed, labeled HSQ-M. The substrates were heat-treated (500 °C, 2 h) to harden the micropillar pattern further. The FTO precursor solution comprised 1.35 and 1.0 × 10^−3^ M of SnCl_4_ and NH_4_F, respectively, in deionized (DI) water. FTO was deposited by performing mist-CVD in a heating chamber at 450 °C with N_2_ as the carrier gas.

### 2.2. Synthesis of Hematite Nanorods

Based on an earlier study, hematite nanorods were prepared using a simple hydrothermal synthesis method [20]. First, 2.5 × 2.5 cm^2^ FTO substrates were cleaned by performing ultrasonication in acetone, ethanol, and DI water for 20 min. Subsequently, UV-ozone treatment was conducted on the FTO substrates for 60 min. The substrates were vertically arranged in a Teflon tube with the precursors of 0.3 M of FeCl_3_·6H_2_O and 2 M of NaNO_3_, in 50 mL of DI water. The Teflon tube was heated at 90 °C for 6 h in a SUS reactor, and then cooled to a temperature of 25 °C. A layer of FeOOH nanorods was fabricated on top of the FTO, and undesirable precipitates on the substrates were removed by rinsing with DI water. The FeOOH nanorods were then dried at 25 °C. To convert FeOOH to Fe_2_O_3_, annealing was performed at 550 °C for 2 h and 750 °C for 20 min. The photoassisted electrodeposition method was used for Co–Pi decoration [21,22]. Co–Pi clusters were deposited as an OER layer on the Fe_2_O_3_ nanorod array by performing photoassisted electrodeposition under illumination (100 mW cm^−2^, AM 1.5 G) at 1.1 V vs. Ag/AgCl for 20 min. The electrolyte was a 0.1 M potassium phosphate buffer solution with 0.5 × 10^−3^ M cobalt chloride hexahydrate.

### 2.3. Characterization

Field-emission scanning electron microscopy (FESEM, SU-8100, Hitachi, Japan), atomic force microscopy (AFM, XE-100, Park Systems, South Korea), and high-resolution transmission electron microscopy (HRTEM, Talos F200X, ThermoFisher Scientific, Waltham, Massachusetts, USA) were employed to study the morphology of the micropillar pattern and the Fe_2_O_3_ photoanodes. A Rigaku SmartLab X-ray diffractometer (Rigaku, Japan) with Cu Kα radiation was used to obtain the X-ray diffraction (XRD) patterns. X-ray photoelectron spectroscopy (XPS, Nexsa XPS system, ThermoFisher Scientific, Waltham, Massachusetts, USA) was performed to analyze the elemental composition. A UV–vis spectrophotometer (SolidSpec-3700, Shimadzu, Japan) was used to measure the transmittance, absorption, and reflectance spectra.

### 2.4. PEC Measurements

The PEC performance of the photoanodes was measured under irradiation (100 mW cm^−2^, AM 1.5 G). *V_RHE_* was calculated as follows [23]:

*V_RHE_* = *V_Ag/AgCl_* + 0.1976 V + 0.0591 pH (1)

where *V_RHE_* is the potential vs. the RHE electrode and *V_Ag/AgCl_* is the potential vs. a Ag/AgCl electrode. The prepared photoanode, Pt coil, and Ag/AgCl electrode were used as the working, counter, and reference electrodes, respectively. The wetted area of the prepared sample was 0.5 × 0.5 cm^2^. The incident photon-to-current efficiency (IPCE) was calculated as follows:

*IPCE* = (1240 × *I_SC_*(A))/(*λ*(nm) × *P*(W)) (2)

where *I_SC_* is the photocurrent density, *λ* is the incident light wavelength, and *P*(W) is the measured irradiance. The IPCE was measured at 1.23 V_RHE_.

Both instances listed above used 1 M of NaOH (pH 13.6) electrolyte.

## 3. Results

### 3.1. Characterization of the Fabricated FTO-M

**Figure 1:**
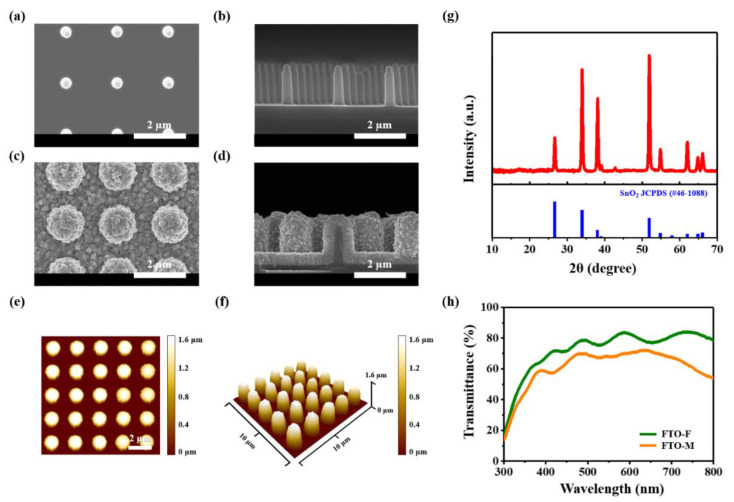
Scanning electron microscopy (SEM) images acquired from the fabrication procedure: (**a**,**b**) HSQ-M. (**c**,**d**) FTO-M. (**e**,**f**) AFM images of FTO-M. (**g**) X-ray diffraction (XRD) spectra of FTO-M. (**h**) Transmittance spectra of FTO-F and FTO-M.

SEM images of the samples produced in each procedure are shown in Figure 1a–d. A periodic array of HSQ-M with a height, diameter, and period of 1.9 μm, 500 nm, and 2.0 μm, respectively, was formed by direct printing (Figure 1a,b). A 450 nm thick FTO film was uniformly deposited on the directly printed HSQ-M (Figure 1c,d). Figure S1 shows the SEM images of the flat FTO, labeled FTO-F, with the same FTO layer thickness of 450 nm. The 2D and 3D AFM images of FTO-M are shown in Figure 1e,f, respectively. The surface area of FTO-F is 134.8 μm^2^ per 10 × 10 μm^2^ (Figure S2). In contrast, the surface area of FTO-M at 233.8 μm^2^ per 10 × 10 μm^2^ is 1.73-fold larger than that of FTO-F. The crystal phase was determined by performing XRD (Figure 1g). Based on the major peaks in the obtained XRD spectra, tetragonal SnO_2_ (JCPDS No. 46-1088) was identified with peaks at 2θ = 26.6°, 33.9°, 38.0°, and 51.8°, corresponding to the (110), (101), (200), and (211) tetragonal SnO_2_ planes, respectively, suggesting the successful fabrication of FTO.

The optical properties of FTO-M and FTO-F were compared using the UV–Vis transmittance spectra (Figure 1h). The transmittance of FTO-M was 11.6% lower on average than that of FTO-F for the entire wavelength range. This largely constant difference in the transmittance is due to the additional light absorption by the FTO layer on the side of the micropillar pattern.

### 3.2. Characterization of the Fabricated Fe_2_O_3_/Co–Pi Photoanode

**Figure 2:**
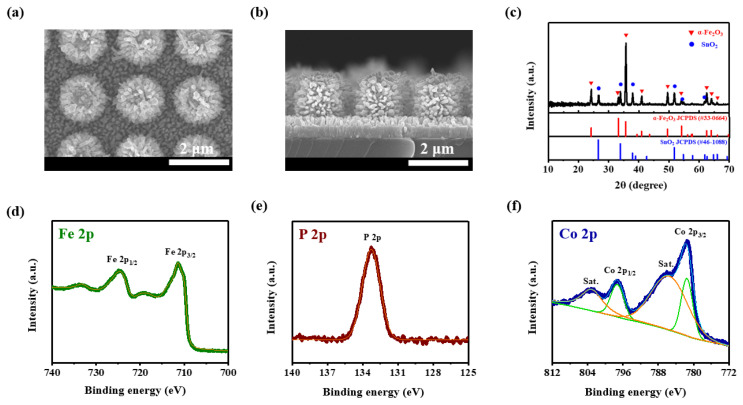
(**a**,**b**) SEM images of the Fe_2_O_3_ nanorods on FTO-M. (**c**) XRD patterns of the FTO-M/Fe_2_O_3_. X-ray photoelectron spectroscopy (XPS) profiles of the Fe_2_O_3_/Co–Pi sample. (**d**) Fe 2p, (**e**) P 2p, (**f**) Co 2p.

The FeOOH nanorods were annealed (Figure 2a,b, Figure S3) to produce Fe_2_O_3_ nanorods. The length and width of the Fe_2_O_3_ nanorods were approximately 350 and 50 nm, respectively. The synthesis of 1D Fe_2_O_3_ nanorods on the 3D micropillar HSQ produces a hierarchical structure with an extremely large surface area. The crystal phase was determined by performing XRD (Figure 2c). Rhombohedral α-Fe_2_O_3_ (JCPDS No. 33-0664) and tetragonal SnO_2_ (JCPDS No. 46-1088) are detected from the main peaks in the XRD patterns. The peaks at 2θ = 24.2°, 33.2°, 35.6°, 40.9°, 49.5°, and 62.5° correspond to the (012), (104), (110), (113), (024), and (214) diffraction planes of rhombohedral α-Fe_2_O_3_, whereas the peaks at 2θ = 26.6°, 33.9°, 38.0°, and 51.8° are attributed to the (110), (101), (200), and (211) tetragonal SnO_2_ planes. No peak related to Co–Pi is observed because of its amorphous nature. Characterization of the chemical state and chemical composition on the surface of the sample was performed using XPS. Figure 2d–f illustrate the narrow-scan XPS profiles of Fe, P, and Co for the prepared sample. The existence of α-Fe_2_O_3_ is further verified by the binding energies at 724.9 and 711.0 eV, which are ascribed to Fe 2p_1/2_ and Fe 2p_3/2_ (Figure 2d) [24]. In Figure S4, the binding energy at 530.2 eV corresponds to the O1s peak, which is attributed to the lattice oxygen(O^2−^) in Fe_2_O_3_. On the other hand, the O1s peak at 531.4 eV is attributed to the surface -OH groups. The spectrum presented in Figure 2e shows a binding energy of 133.0 eV, corresponding to a single peak of P 2p that is attributed to the phosphate in the electrodeposited Co–Pi clusters. Furthermore, the binding energies at 797.3 and 781.6 eV correspond to Co 2p_1/2_ and Co 2p_3/2_, in the Co 2p region (Figure 2f). The binding energies of the two shakeup satellite peaks (Sat.) at 785.7 and 803.1 eV are assigned to the oxidized Co in the +2 oxidation state (Co^2+^). An increase in the Co^2+^/Co^3+^ ratio of the sample is observed when the peak shifts to a higher binding energy [25,26]. The oxidation number of the cobalt ions is assumed to change cyclically (Co^2+/3+^ → Co^3+^ → Co^2+/3+^) during the oxidation of water [27,28]. Therefore, the ratio of Co^2+^/Co^3+^ directly after deposition does not have a significant effect on the overall catalytic ability compared to either the nucleation density or the total number of Co–Pi clusters, thereby verifying the formation of Co–Pi on the Fe_2_O_3_ sample [29].

**Figure 3:**
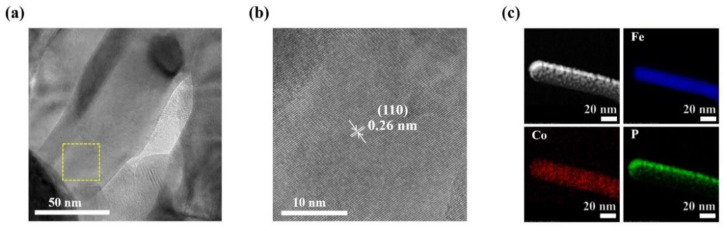
(**a**) Low-resolution transmission electron microscopy (TEM) image of the Fe_2_O_3_ nanorod. (**b**) High-resolution TEM image of the dashed yellow square in (**a**). (**c**) TEM-energy dispersive spectroscopy (EDS) mapping images of Fe_2_O_3_/Co–Pi.

Figure 3 shows the TEM images used to determine the crystallinity of Fe_2_O_3_. Figure 3a illustrates a low-resolution TEM image of the Fe_2_O_3_ nanorod. The HRTEM image (Figure 3b) shows the crystalline structure of the Fe_2_O_3_ nanorod. The lattice spacing, measured as 0.26 nm, is attributed to the Fe_2_O_3_ (110) plane. In Figure 3c, energy-dispersive X-ray spectrometry (EDS) elemental mapping was used to determine the presence of Fe, Co, and P; these elements were all found to be uniformly distributed throughout the sample.

### 3.3. Optical Properties of the Hematite Nanorod on the Micropillar FTO Photoanode

**Figure 4:**
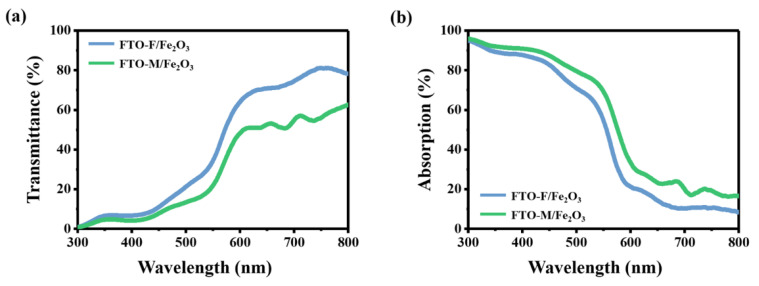
Optical properties of FTO-M/Fe_2_O_3_ and FTO-F/Fe_2_O_3_: (**a**) Transmittance and (**b**) Absorption.

The optical properties of the Fe_2_O_3_ nanorods on FTO-M (FTO-M/Fe_2_O_3_) and Fe_2_O_3_ nanorods on FTO-F (FTO-F/Fe_2_O_3_) were compared using UV–Vis transmittance and absorption spectra (Figure 4). The transmittance of FTO-M/Fe_2_O_3_ is on average 15.1% lower than that of FTO-F/Fe_2_O_3_ throughout the wavelength region. The uniform light-scattering effect is due to the periodic micropillar array and the Fe_2_O_3_ nanorods that grow horizontally on the side of the micropillar and act as a thick photoactive layer to lower the transmittance of the photoanode. Figure S5 presents the reflectance spectra of FTO-M/Fe_2_O_3_ and FTO-F/Fe_2_O_3_. The average reflectance of FTO-M/Fe_2_O_3_ is 1.03% less than that of FTO-F/Fe_2_O_3_ in the 300–500 nm wavelength region. The small difference between these reflectance values is attributed to the random light scattering by the Fe_2_O_3_ nanorods of both samples. The absorption of FTO-M/Fe_2_O_3_ is higher than that of FTO-F/Fe_2_O_3_ in all wavelength ranges. Not only do the Fe_2_O_3_ nanorods grown horizontally on the side of the micropillar increase the absorption of FTO-M/Fe_2_O_3_, but the light-scattering effect of the periodic micropillar array and randomly arranged Fe_2_O_3_ nanorods also contribute greatly toward increasing the absorption of the sample [30,31].

The absorption efficiency (*η_abs_*) was calculated as follows [32,33]:

*η_abs_ = J_abs_/J_max_* (3)

where *J_max_* is the integral value at AM, calculated by the trapezoidal integration of the AM 1.5 G solar spectral irradiance (in 1 nm increments) up to the absorption wavelength, and *J_abs_* is the integral value of the product, calculated by the stepwise multiplication of the absorption and spectral irradiance in the trapezoidal integration up to the absorption edge. The optical bandgap of 2.11 eV and absorption wavelength of 587 nm were calculated using the Tauc plot (Figure S6); thus, *J_max_* was 12.25 mA cm^−2^. For FTO-F/Fe_2_O_3_, *J_abs_* was 8.37 mA cm^−2^ and *η_abs_* was 68.3%. For FTO-M/Fe_2_O_3_, *J_abs_* was 9.45 mA cm^−2^ and *η_abs_* was 77.5%, which was 9.2% higher than that of FTO-F/Fe_2_O_3_. Therefore, the light absorption efficiency was enhanced by the periodic micropatterns and random Fe_2_O_3_ nanorods.

### 3.4. PEC Performance of the Micropillar-Patterned Hematite Nanorod Photoanode

**Figure 5:**
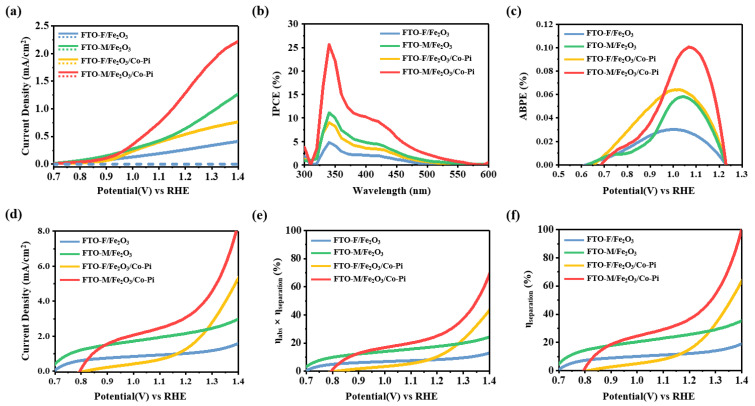
Photoelectrochemical (PEC) performance characteristics of FTO-F/Fe_2_O_3_, FTO-M/Fe_2_O_3_, FTO-F/Fe_2_O_3_/Co–Pi, and FTO-M/Fe_2_O_3_/Co–Pi. (**a**) J–V curves in 1 M of NaOH under illumination. (**b**) IPCE measured at 1.23 V_RHE_. (**c**) ABPE calculated from J–V curves. (**d**) J_H₂O₂_–V curves measured in 0.1 M of NaOH with 0.5 M of hydrogen peroxide (H_2_O_2_) under illumination. (**e**) Absorption efficiency multiplied by the charge separation efficiency (η*_abs_* × η_separation_). (**f**) Charge separation efficiency (η_separation_).

The current density–voltage (J–V) curves of FTO-F/Fe_2_O_3_, FTO-M/Fe_2_O_3_, FTO-F/Fe_2_O_3_/Co–Pi, and FTO-M/Fe_2_O_3_/Co–Pi, measured in 1 M NaOH under solar irradiance (100 mW cm^−2^, AM 1.5 G), are illustrated in Figure 5a. The photocurrent density of FTO-F/Fe_2_O_3_ is 0.29 mA cm^−2^, whereas that of FTO-M/Fe_2_O_3_ is 2.6 times higher, at 0.75 mA cm^−2^. This increased PEC efficiency is attributed to the expanded specific area resulting from the hierarchical structure and multiple light scattering from the micropillar FTO and Fe_2_O_3_ nanorods. The mechanism of the Co–Pi catalyst can be explained by its cyclic reactions of cobalt ions. The Co–Pi layer captures the holes from the valence band of Fe_2_O_3_ and oxidizes cobalt ions. These Co species can oxidize water more effectively than bare Fe_2_O_3_ surfaces [34]. The PEC efficiency is also enhanced by the decoration of the Co–Pi catalyst, due to the improvement in the surface water oxidation activity. Thus, the photocurrent densities generated by FTO-F/Fe_2_O_3_/Co–Pi and FTO-M/Fe_2_O_3_/Co–Pi increase to 0.59 and 1.51 mA cm^−2^, respectively. Transient photocurrent measurement (Figure S7) was also employed to demonstrate the stability of Fe_2_O_3_ nanorods and Co–Pi catalyst.

The IPCE was determined to analyze the wavelength-related PEC performance (Figure 5b). FTO-M/Fe_2_O_3_/Co–Pi exhibits higher IPCE values than the other photoanodes at all measured wavelengths. The highest IPCE values for FTO-F/Fe_2_O_3_, FTO-F/Fe_2_O_3_/Co–Pi, FTO-M/Fe_2_O_3_, and FTO-M/Fe_2_O_3_/Co–Pi are 4.84, 9.03, 11.1, and 25.7%, respectively. This result indicates that the micropillar structure and Co–Pi catalyst have a remarkable influence on the charge kinetics at the surface and on PEC efficiency. Further, in Figure S8, the electrochemical impedance spectra (Nyquist plots) show the decrease of interface resistance between the electrolyte and the photoanode, which is R2 of the circuit. The decrease of R2 could be interpreted as due to the enlarged surface area and the enhanced surface oxidation reaction by Co–Pi catalyst.

The PEC efficiency of the photoanodes was quantitatively analyzed using the applied bias photon-to-current efficiency (ABPE). The maximum photoconversion efficiency of FTO-M/Fe_2_O_3_/Co–Pi was 0.101% at 1.07 V_RHE_, whereas that of FTO-F/Fe_2_O_3_ was only 0.0302% at 1.00 V_RHE_ (Figure 5c). 

The effect of surface structuring and Co–Pi cluster decoration was further investigated by adding 0.5 M of an H_2_O_2_ solution to 1 M of NaOH (Figure 5d). The H_2_O_2_ solution acted as a hole scavenger and suppressed surface recombination; hence, it was used to measure the maximum PEC properties without an injection barrier, by assuming charge transfer efficiency (*η_transfer_*) to be 100% [32,35]. The obtained photocurrent densities were 1.05, 1.62, 2.24, and 3.39 mA cm^−2^ for FTO-F/Fe_2_O_3_, FTO-F/Fe_2_O_3_/Co–Pi, FTO-M/Fe_2_O_3_, and FTO-M/Fe_2_O_3_/Co–Pi, respectively. From Figure 5e,f, 𝜂*_abs_* × 𝜂_separation_ and 𝜂_separation_ were calculated as follows [36,37,38]:

𝐽_H₂O₂_ (𝜂_transfer_ ≈ 100%)  =  𝐽*_max_* × 𝜂*_abs_* × 𝜂_separation_, (4)

𝜂*_abs_* × 𝜂_separation_ = 𝐽_H₂O₂_/𝐽*_max_*, (5)

𝜂_separation_  = 𝐽_H₂O₂_/(𝐽*_max_* × 𝜂*_abs_*). (6)

The *J_max_* of Fe_2_O_3_ was 12.2 mA cm^−2^ (300–587 nm). The calculated 𝜂*_abs_* × 𝜂_separation_ values were 8.68, 20.2, 26.7, and 41.0% for FTO-F/Fe_2_O_3_, FTO-F/Fe_2_O_3_/Co–Pi, FTO-M/Fe_2_O_3_, and FTO-M/Fe_2_O_3_/Co–Pi, respectively. These results confirm that 𝜂_separation_ increases because the 1D nanorods on the 3D micropillar-patterned FTO significantly increase the active area to produce multiple light-scattering effects, whereas Co–Pi alleviates surface charge recombination and acts as a passivation layer.

## 4. Conclusions

In this study, we employed a simple, low-cost, and large-area-fabrication approach for FTO photoanodes. We thus fabricated 3D branched Fe_2_O_3_ nanorods on a micropillar FTO with an effective PEC water splitting performance with the electrodeposition of Co–Pi clusters and direct printing. The *η_abs_* of FTO-F/Fe_2_O_3_ increased by 9.2% compared to that of FTO-M/Fe_2_O_3_ because of the uniform and random light scattering induced by the periodic micropillar FTO and Fe_2_O_3_ nanorods, respectively. In addition, the surface area and separation efficiency were greatly enhanced because of the unique structure of the 1D Fe_2_O_3_ nanorods that grew on the 3D micropillar array. The additional decoration of a Co–Pi catalyst oxidized water more effectively than the bare Fe_2_O_3_ surface and yielded a photocurrent density of 1.51 mA cm^−2^. This study illustrates the critical role of micro-nanoscale structural engineering and surface oxidation catalysts in enhancing the PEC performance of hematite, thereby paving the way for low-cost solar-powered water splitting applications.

## Data Availability

The data used and or analyzed during the current study are available from the corresponding author upon request.

## Supplementary Materials

The following supporting information can be downloaded at: https://www.mdpi.com/article/10.3390/nano12203664/s1. Figure S1: SEM images of FTO-F, Figure S2: AFM results for FTO-F, Figure S3: SEM images of the Fe_2_O_3_ nanorods on FTO-F, Figure S4: XPS profile of O1s from the Fe_2_O_3_/Co–Pi sample, Figure S5: Reflectance of flat Fe_2_O_3_ and Fe_2_O_3_-M, Figure S6: Tauc plot of flat Fe_2_O_3_, Figure S7: Transient photocurrents of FTO-F/Fe_2_O_3_, FTO-M/Fe_2_O_3_, FTO-F/Fe_2_O_3_/Co–Pi, and FTO-M/Fe_2_O_3_/Co–Pi in 1 M of NaOH under illumination, Figure S8: (a) Nyquist plots from electrochemical impedance spectroscopy (EIS); (b) the equivalent circuit model from the fitting results.

## References

[1] Walter M.G., Warren E.L., McKone J.R., Boettcher S.W., Mi Q., Santori E.A., Lewis N.S. (2010). Solar water splitting cells. Chem. Rev..

[2] Reece S.Y., Hamel J.A., Sung K., Jarvi T.D., Esswein A.J., Pijpers J.J.H., Nocera D.G. (2011). Wireless solar water splitting using silicon-based semiconductors and earth-abundant catalysts. Science.

[3] Landman A., Dotan H., Shter G.E., Wullenkord M., Houaijia A., Maljusch A., Grader G.S., Rothschild A. (2017). Photoelectrochemical water splitting in separate oxygen and hydrogen cells. Nat. Mater..

[4] Du C., Yang X., Mayer M.T., Hoyt H., Xie J., McMahon G., Bischoping G., Wang D. (2013). Hematite-based water splitting with low turn-on voltages. Angew. Chem..

[5] Hisatomi T., Kubota J., Domen K. (2014). Recent advances in semiconductors for photocatalytic and photoelectrochemical water splitting. Chem. Soc. Rev..

[6] Kim J.Y., Youn D.H., Kang K., Lee J.S. (2016). Highly conformal deposition of an ultrathin FeOOH layer on a hematite nanostructure for efficient solar water splitting. Angew. Chem..

[7] Wang W., Jin C., Qi L. (2018). Hierarchical CdS nanorod@SnO2 nanobowl arrays for efficient and stable photoelectrochemical hydrogen generation. Small.

[8] Pan Q., Zhang H., Yang Y., Cheng C. (2019). 3D Brochosomes-like TiO_2_/WO_3_/BiVO_4_ arrays as photoanode for photoelectrochemical hydrogen production. Small.

[9] Quang N.D., Hu W., Chang H.S., Van P.C., Viet D.D., Jeong J.R., Seo D.B., Kim E.T., Kim C., Kim D. (2021). Fe_2_O_3_ hierarchical tubular structure decorated with cobalt phosphide (CoP) nanoparticles for efficient photoelectrochemical water splitting. Chem. Eng. J..

[10] Wang Z., Zhu H., Tu W., Zhu X., Yao Y., Zhou Y., Zou Z. (2022). Host/guest nanostructured photoanodes integrated with targeted enhancement strategies for photoelectrochemical water splitting. Adv. Sci. (Weinh).

[11] Wang L., Palacios-Padrós A., Kirchgeorg R., Tighineanu A., Schmuki P. (2014). Enhanced photoelectrochemical water splitting efficiency of a hematite-ordered Sb:SnO_2_ host-guest system. ChemSusChem.

[12] Resasco J., Zhang H., Kornienko N., Becknell N., Lee H., Guo J., Briseno A.L., Yang P. (2016). TiO_2_/BiVO_4_ nanowire heterostructure photoanodes based on type II band alignment. ACS Cent. Sci..

[13] Zhou L., Zhao C., Giri B., Allen P., Xu X., Joshi H., Fan Y., Titova L.V., Rao P.M. (2016). High light absorption and charge separation efficiency at low applied voltage from Sb-doped SnO_2_/BiVO_4_ core/shell nanorod-array photoanodes. Nano Lett..

[14] Garcia-Torregrosa I., Wijten J.H.J., Zanoni S., Oropeza F.E., Hofmann J.P., Hensen E.J.M., Weckhuysen B.M. (2019). Template-free nanostructured fluorine-doped tin oxide scaffolds for photoelectrochemical water splitting. ACS Appl. Mater. Interfaces.

[15] Wang Z., Nguyen T.D., Yeo L.P., Tan C.K., Gan L., Tok A.I.Y. (2020). Periodic FTO IOs/CdS NRs/CdSe clusters with superior light scattering ability for improved photoelectrochemical performance. Small.

[16] Wang Z., Li X., Ling H., Tan C.K., Yeo L.P., Grimsdale A.C., Tok A.I.Y. (2018). 3D FTO/FTO-nanocrystal/TiO_2_ composite inverse opal photoanode for efficient photoelectrochemical water splitting. Small.

[17] Li J., Qiu Y., Wei Z., Lin Q., Zhang Q., Yan K., Chen H., Xiao S., Fan Z., Yang S. (2014). A three-dimensional hexagonal fluorine-doped tin oxide nanocone array: A superior light harvesting electrode for high performance photoelectrochemical water splitting. Energy Environ. Sci..

[18] Ju S., Kang H., Jun J., Son S., Park J., Kim W., Lee H. (2021). Periodic micropillar-patterned FTO/BiVO_4_ with superior light absorption and separation efficiency for efficient PEC performance. Small.

[19] Ju S., Seok H.J., Jun J., Huh D., Son S., Kim K., Kim W., Baek S., Kim H.K., Lee H. (2020). Fully blossomed WO_3_/BiVO_4_ structure obtained via active facet engineering of patterned FTO for highly efficient water splitting. Appl. Catal. B.

[20] Jeon T.H., Moon G.H., Park H., Choi W. (2017). Ultra-efficient and durable photoelectrochemical water oxidation using elaborately designed hematite nanorod arrays. Nano Energy.

[21] Jeon T.H., Choi W., Park H. (2011). Cobalt-phosphate complexes catalyze the photoelectrochemical water oxidation of BiVO_4_ electrodes. Phys. Chem. Chem. Phys..

[22] Chai X., Zhang H., Pan Q., Bian J., Chen Z., Cheng C. (2019). 3D ordered urchin-like TiO_2_@Fe_2_O_3_ arrays photoanode for efficient photoelectrochemical water splitting. Appl. Surf. Sci..

[23] Liu C., Wang F., Zhang J., Wang K., Qiu Y., Liang Q., Chen Z. (2018). Efficient photoelectrochemical water splitting by g-C_3_N_4_/TiO_2_ nanotube array heterostructures. Nano Micro Lett..

[24] Jia L., Xie J., Guo C., Li C.M. (2015). Modification of a thin layer of α-Fe_2_O_3_ onto a largely voided TiO_2_ nanorod array as a photoanode to significantly improve the photoelectrochemical performance toward water oxidation. RSC Adv..

[25] Wang Y., Zhou T., Jiang K., Da P., Peng Z., Tang J., Kong B., Cai W.B., Yang Z., Zheng G. (2014). Reduced mesoporous Co_3_O_4_ nanowires as efficient water oxidation electrocatalysts and supercapacitor electrodes. Adv. Energy Mater..

[26] Ai G., Mo R., Li H., Zhong J. (2015). Cobalt phosphate modified TiO_2_ nanowire arrays as co-catalysts for solar water splitting. Nanoscale.

[27] McDonald K.J., Choi K.S. (2011). Photodeposition of co-based oxygen evolution catalysts on α-Fe_2_O_3_ photoanodes. Chem. Mater..

[28] Ai L., Niu Z., Jiang J. (2017). Mechanistic insight into oxygen evolution electrocatalysis of surface phosphate modified cobalt phosphide nanorod bundles and their superior performance for overall water splitting. Electrochim. Acta.

[29] Wang Y., Chen D., Wang S., Liang J., Qin L., Sun X., Huang Y. (2019). Photoassisted electrodeposition of cobalt-phosphate cocatalyst on BiFeO_3_ thin film photoanode for highly efficient photoelectrochemical performances of water oxidation. J. Electrochem. Soc..

[30] Raut H.K., Ganesh V.A., Nair A.S., Ramakrishna S. (2011). Anti-reflective coatings: A critical, in-depth review. Energy Environ. Sci..

[31] Zhou T., Wang J., Chen S., Bai J., Li J., Zhang Y., Li L., Xia L., Rahim M., Xu Q. (2020). Bird-nest structured ZnO/TiO_2_ as a direct Z-scheme photoanode with enhanced light harvesting and carriers kinetics for highly efficient and stable photoelectrochemical water splitting. Appl. Catal. B.

[32] Dotan H., Sivula K., Grätzel M., Rothschild A., Warren S.C. (2011). Probing the photoelectrochemical properties of hematite (α-Fe_2_O_3_) electrodes using hydrogen peroxide as a hole scavenger. Energy Environ. Sci..

[33] Nair V., Perkins C.L., Lin Q., Law M. (2016). Textured nanoporous Mo:BiVO_4_ photoanodes with high charge transport and charge transfer quantum efficiencies for oxygen evolution. Energy Environ. Sci..

[34] Eftekharinia B., Moshaii A., Dabirian A., Vayghan N.S. (2017). Optimization of charge transport in a Co-Pi modified hematite thin film produced by scalable electron beam evaporation for photoelectrochemical water oxidation. J. Mater. Chem. A.

[35] Li L., Liu C., Zhang H., Liang P., Mitsuzaki N., Chen Z. (2018). Synergistic effect of Ti(OBu)_4_ and annealing regime on the structure, morphology and photoelectrochemical response of α-Fe_2_O_3_ photoanode. Electrochim. Acta.

[36] Yi S.S., Wulan B.R., Yan J.M., Jiang Q. (2019). Highly efficient photoelectrochemical water splitting: Surface modification of cobalt-phosphate-loaded Co_3_O_4_/Fe_2_O_3_ p–n heterojunction nanorod arrays. Adv. Funct. Mater..

[37] Bu X., Gao Y., Zhang S., Tian Y. (2019). Amorphous cerium phosphate on P-doped Fe_2_O_3_ nanosheets for efficient photoelectrochemical water oxidation. Chem. Eng. J..

[38] Chen D., Liu Z., Guo Z., Ruan M., Yan W. (2019). 3D branched Ca-Fe_2_O_3_/Fe_2_O_3_ decorated with Pt and co-pi: Improved charge-separation dynamics and photoelectrochemical performance. ChemSusChem.

