# Supplementary material for: Hierarchical Co–Pi Clusters/Fe2O3 Nanorods/FTO Micropillars 3D Branched Photoanode for High-Performance Photoelectrochemical Water Splitting
Source: Nanomaterials (Basel). 2022 Oct 18;12(20):3664. doi: 10.3390/nano12203664 (PMC9611282; doi:10.3390/nano12203664)
Supplement: Supplementary file 1 [file nanomaterials-12-03664-s001.zip › nanomaterials-1960756-supplementary.pdf]

Supplementary Materials

Hierarchical Co–Pi Clusters/Fe_2O_3 Nanorods/FTO Micropillars 3D Branched Photoanode for High-Performance Photoelectrochemical Water Splitting

Nakhyun Kim [1,†], Sucheol Ju [2,†], Jisung Ha [2], Hojung Choi [2], Hansang Sung [2] and Heon Lee [1,2,*]

[1] Department of Semiconductor Systems Engineering, Korea University, Anam-ro 145, Sungbuk-Gu, Seoul 136-701, Korea

[2] Department of Materials Science and Engineering, Korea University, Anam-ro 145, Sungbuk-Gu, Seoul 136-701, Korea

* Correspondence: heonlee@korea.ac.kr

† These authors contributed equally to this work.

Supplementary Materials

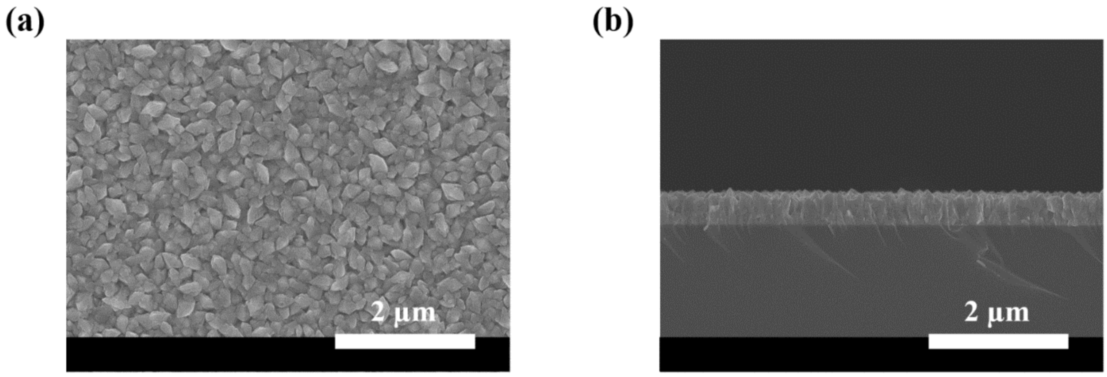

Figure S1. SEM images of FTO-F.

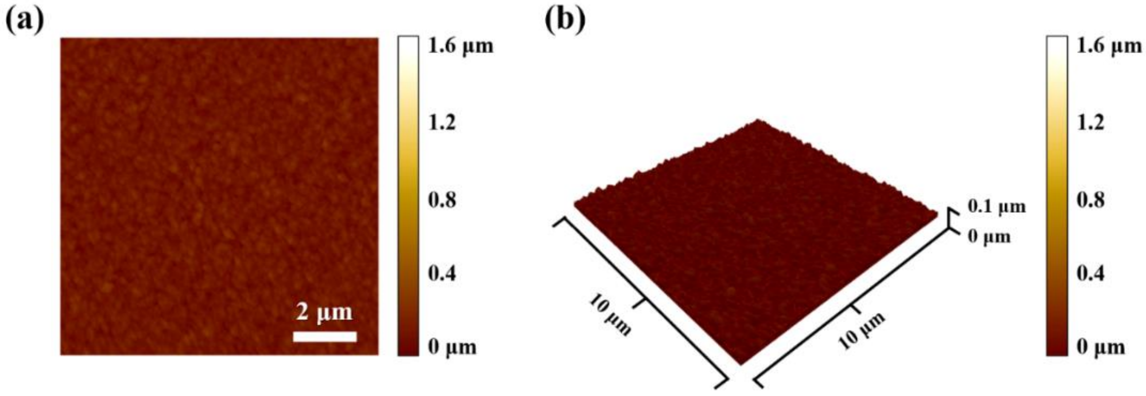

Figure S2. AFM results for FTO-F.

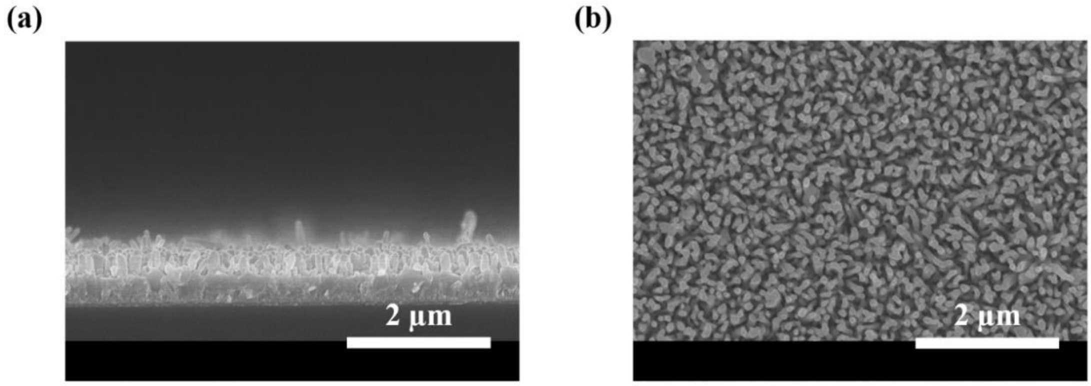

Figure S3. SEM images of the Fe_2O_3 nanorods on FTO-F.

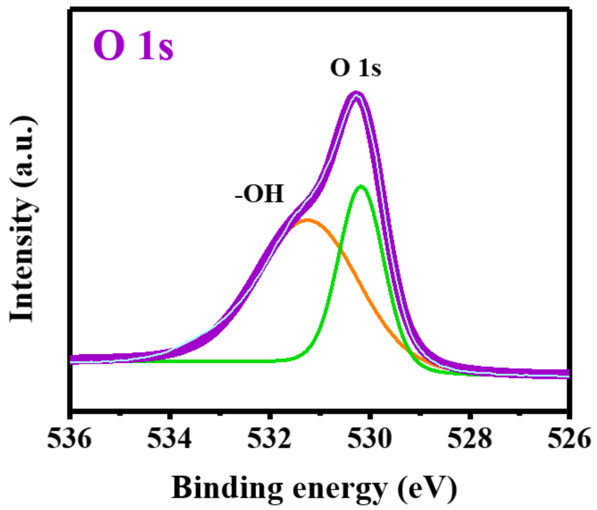

Figure S4. XPS profile of O1s from the Fe_2O_3/Co–Pi sample.

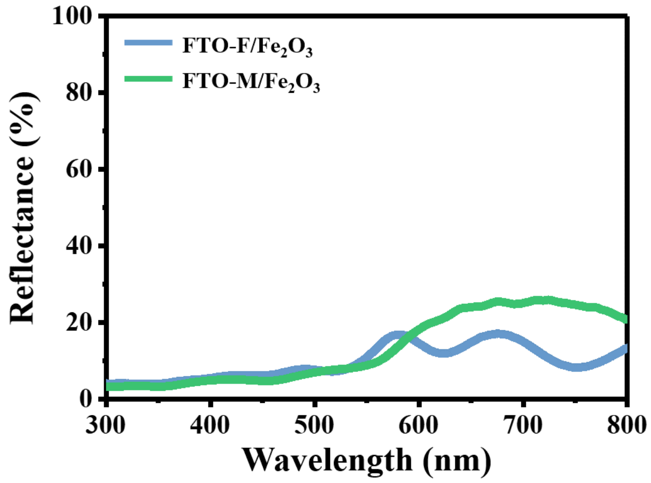

Figure S5. Reflectance of flat Fe_2O_3 and Fe_2O_3-M.

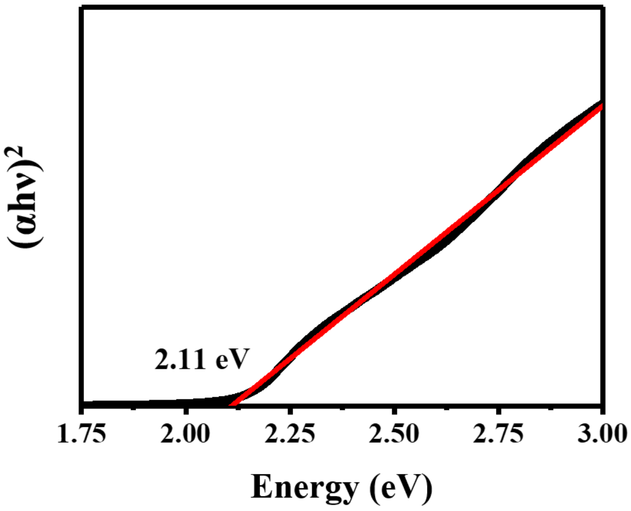

Figure S6. Tauc plot of flat Fe_2O_3.

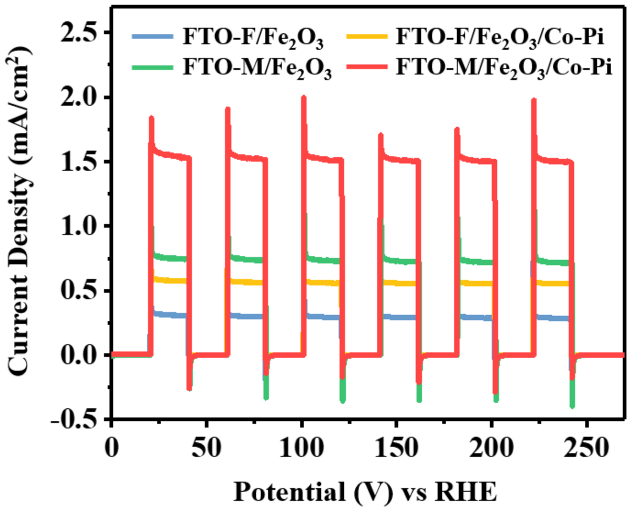

Figure S7. Transient photocurrents of FTO-F/Fe_2O_3, FTO-M/Fe_2O_3, FTO-F/Fe_2O_3/Co–Pi, and FTO-M/Fe_2O_3/Co–Pi in 1 M of NaOH under illumination.

(a)

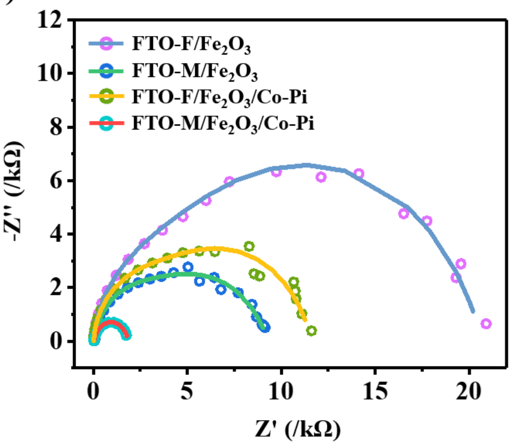

(b)

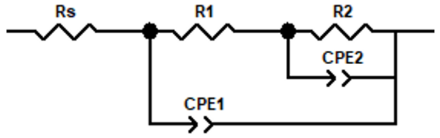

Sample	Rs (Ω)	R1 (Ω)<br>CPE1 (F)	R2 (Ω)<br>CPE2 (F)
FTO-F/Fe_2O_3	32.1	6736<br>1.01×10^{-6}	13900<br>7.25×10^{-6}
FTO-F/Fe_2O_3/Co-Pi	22.1	5043<br>8.20×10^{-7}	6541<br>9.59×10^{-6}
FTO-M/Fe_2O_3	60.0	4055<br>1.34×10^{-6}	5174<br>1.93×10^{-5}
FTO-M/Fe_2O_3/Co-Pi	23.0	20.89<br>2.05×10^{-7}	1813<br>4.18×10^{-6}

Figure S8. (**a**) Nyquist plots from electrochemical impedance spectroscopy (EIS). (**b**) the equivalent circuit model from the fitting results.
